# Supplementary material for: Peritoneal Fluid Cytokines Reveal New Insights of Endometriosis Subphenotypes
Source: Int J Mol Sci. 2020 May 15;21(10):3515. doi: 10.3390/ijms21103515 (PMC7278942; doi:10.3390/ijms21103515)
Supplement: Supplementary file 1 [file ijms-21-03515-s001.zip › Supplementary Figures.pptx]

## Slide 1
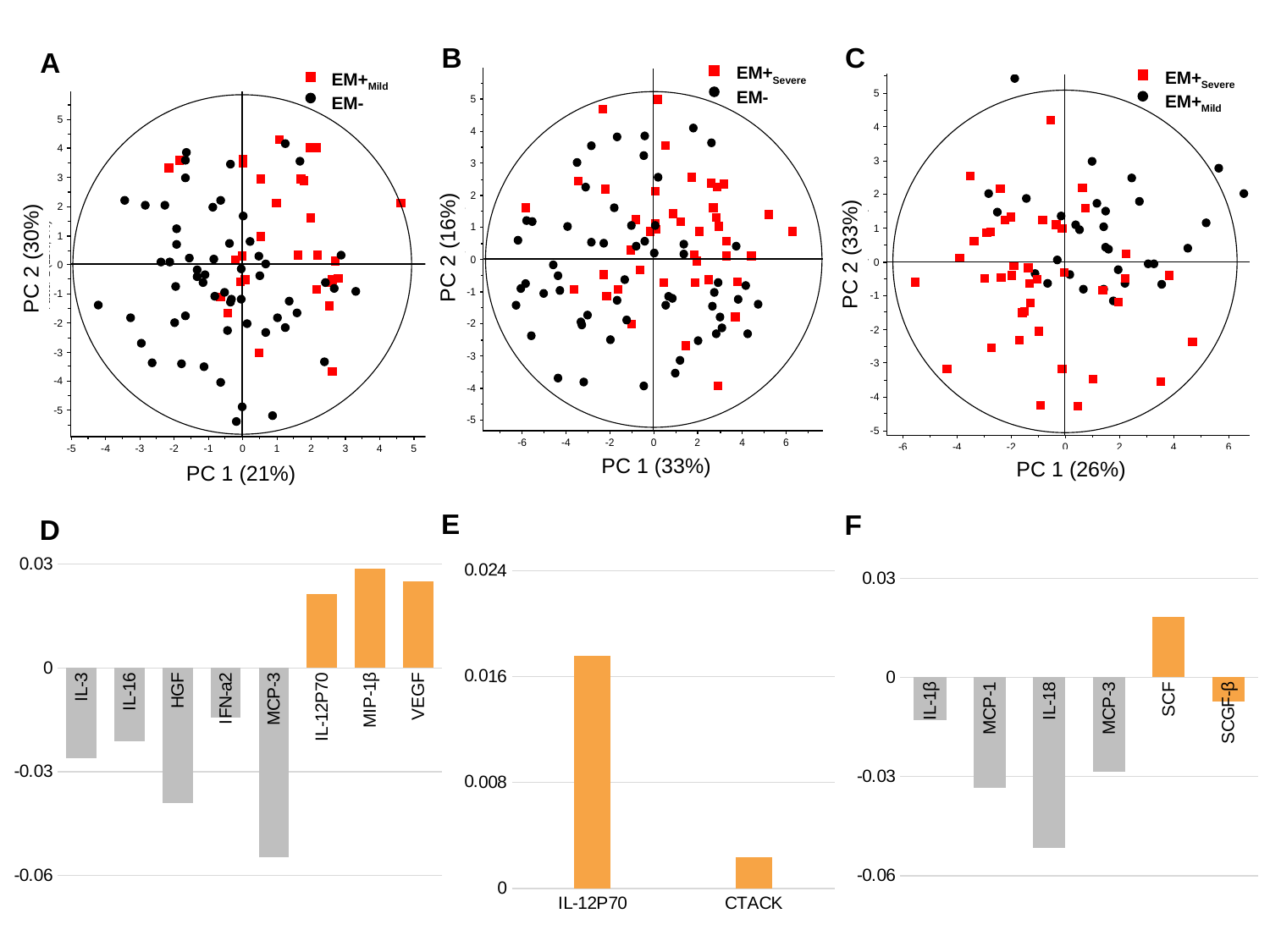

B
C
A
EM+Severe
EM-
EM+Severe
EM+Mild
EM+Mild
EM-
PC 2 (16%)
PC 2 (33%)
PC 2 (30%)
PC 1 (33%)
PC 1 (26%)
PC 1 (21%)
E
F
D
### Chart
| Category | |
|---|---|
| IL-3 | -0.02621042 |
| IL-16 | -0.02113693 |
| HGF | -0.03908136 |
| IFN-a2 | -0.01446582 |
| MCP-3 | -0.05464383 |
| IL-12P70 | 0.0213104 |
| MIP-1β | 0.02857304 |
| VEGF | 0.02494765 |
### Chart
| Category | |
|---|---|
| IL-12P70 | 0.01755654 |
| CTACK | 0.002382925 |
### Chart
| Category | |
|---|---|
| IL-1β | -0.01305515 |
| MCP-1 | -0.03336453 |
| IL-18 | -0.05147515 |
| MCP-3 | -0.0286754 |
| SCF | 0.01835497 |
| SCGF-β | -0.007237166 |

## Slide 2
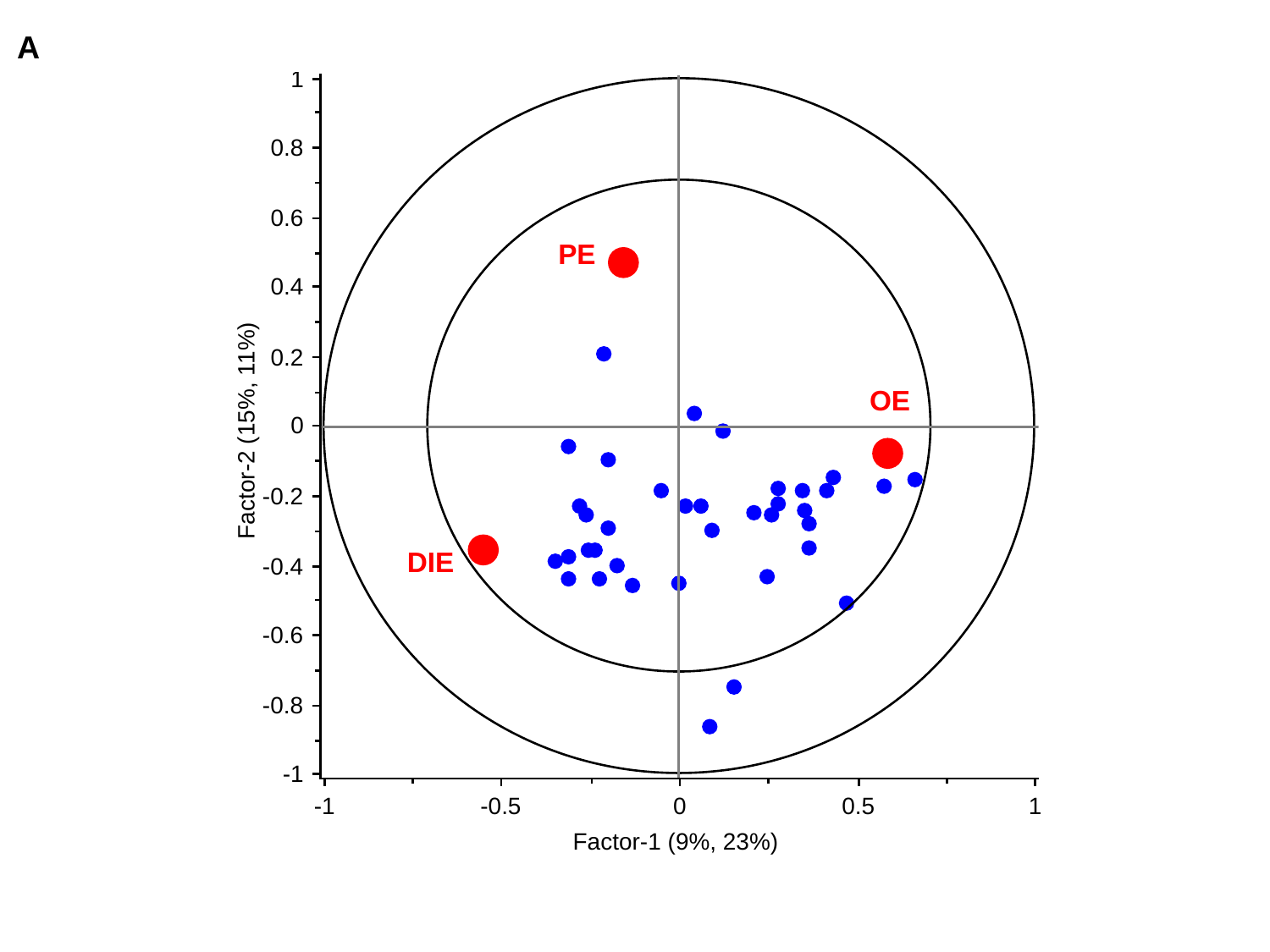

A
PE
OE
DIE
