# Supplementary material for: Peritoneal Fluid Cytokines Reveal New Insights of Endometriosis Subphenotypes
Source: Int J Mol Sci. 2020 May 15;21(10):3515. doi: 10.3390/ijms21103515 (PMC7278942; doi:10.3390/ijms21103515)
Supplement: Supplementary file 1 [file ijms-21-03515-s001.zip › Table S2.pdf]

Table S2. Significantly altered cytokines in endometriosis and disease severity by univariate statistical analysis

All Phase	EM+ vs EM-		$EM+_{Mild}$ vs EM-		$EM+_{Sev}$ vs EM-		$EM+_{Sev}$ vs $EM+_{Mild}$	
	Fold Change	*P*-value	Fold Change	*P*-value	Fold Change	*P*-value	Fold Change	*P*-value
**IFN-α2**	0.95	0.03	0.95	0.03	0.97	0.29	1.02	0.54
**IL-1β**	1.80	0.04	1.80	0.04	1.18	0.24	0.65	0.11
**IL-3**	0.89	0.04	0.89	0.04	0.93	0.46	1.04	0.56
**IL-12p70**	1.20	0.24	1.20	0.24	1.38	0.02	1.15	0.30
**IL-16**	0.96	0.01	0.96	0.01	0.98	0.38	1.02	0.26
**IL-18**	0.79	0.42	0.79	0.42	1.39	0.08	1.75	0.02
**MCP-1**	0.95	0.43	0.95	0.43	1.10	0.11	1.15	0.01
**MCP-3**	0.85	0.01	0.85	0.01	0.97	0.84	1.14	0.05
**VEGF-A**	1.00	0.13	1.00	0.13	1.10	0.05	1.10	0.72

Proliferative Phase	EM+ vs EM-		$EM+_{Mild}$ vs EM-		$EM+_{Sev}$ vs EM-		$EM+_{Sev}$ vs $EM+_{Mild}$	
	Fold Change	*P*-value	Fold Change	*P*-value	Fold Change	*P*-value	Fold Change	*P*-value
**CTACK**	1.08	0.02	1.08	0.02	1.03	0.29	0.96	0.15
**IFN-α2**	0.94	0.05	0.94	0.05	0.95	0.04	1.01	0.73
**IL-12p70**	1.77	0.01	1.77	0.01	2.11	0.00	1.19	0.36
**IL-18**	1.22	0.69	1.22	0.69	2.03	0.02	1.66	0.11
**IP-10**	1.04	0.19	1.04	0.19	1.07	0.02	1.03	0.41
**MCP-1**	0.97	0.74	0.97	0.74	1.16	0.04	1.20	0.02
**MCP-3**	0.79	0.02	0.79	0.02	1.01	0.89	1.27	0.01
**MIP-1α**	0.78	0.16	0.78	0.16	0.69	0.03	0.88	0.60
**VEGF-A**	1.18	0.02	1.18	0.02	1.32	0.01	1.12	0.80

*No differences in cytokine levels in secretory phase
