# Supplementary material for: Peritoneal Fluid Cytokines Reveal New Insights of Endometriosis Subphenotypes
Source: Int J Mol Sci. 2020 May 15;21(10):3515. doi: 10.3390/ijms21103515 (PMC7278942; doi:10.3390/ijms21103515)
Supplement: Supplementary file 1 [file ijms-21-03515-s001.zip › Table S3.pdf]

**Table S3. Significantly altered cytokines in endometriosis subphenotypes by univariate statistical analysis**

| All Phases | OE vs DIE | | PE vs DIE | | OE vs PE | |
|---|---|---|---|---|---|---|
| | Fold Change | *P*-value | Fold Change | *P*-value | Fold Change | *P*-value |
| **IL-1α** | 1.20 | 0.009 | 1.30 | 0.0007 | 1.09 | 0.098 |
| **IL-1β** | 4.09 | 0.004 | 4.56 | 0.0143 | 1.12 | 0.698 |
| **IL-1RA** | 1.32 | 0.001 | 1.14 | 0.2754 | 0.86 | 0.079 |
| **IL-8** | 0.69 | 0.114 | 0.36 | 0.0091 | 0.47 | 0.014 |
| **IL-16** | 0.95 | 0.118 | 0.92 | 0.0192 | 0.97 | 0.397 |
| **MCP-1** | 1.03 | 0.687 | 0.82 | 0.0651 | 0.80 | 0.003 |
| **TNF-α** | 1.50 | 0.003 | 1.19 | 0.2321 | 0.79 | 0.060 |

| Proliferative | OE vs DIE | | PE vs DIE | | OE vs PE | |
|---|---|---|---|---|---|---|
| | Fold Change | *P*-value | Fold Change | *P*-value | Fold Change | *P*-value |
| **IL-1ra** | 0.66 | 0.003 | 0.74 | 0.061 | 1.13 | 0.306 |
| **IL-8** | 1.29 | 0.288 | 3.45 | 0.011 | 2.66 | 0.053 |
| **IL-10** | 0.58 | 0.030 | 0.80 | 0.175 | 1.39 | 0.069 |
| **MCP-1** | 0.94 | 0.452 | 1.22 | 0.175 | 1.30 | 0.022 |
| **TNF-a** | 0.51 | 0.004 | 0.73 | 0.169 | 1.42 | 0.042 |
| **IL-1a** | 0.71 | 0.002 | 0.72 | 0.010 | 1.01 | 0.844 |
| **IL-2Ra** | 1.06 | 0.369 | 1.20 | 0.039 | 1.13 | 0.093 |

| Secretory | OE vs DIE | | PE vs DIE | | OE vs PE | |
|---|---|---|---|---|---|---|
| | Fold Change | *P*-value | Fold Change | *P*-value | Fold Change | *P*-value |
| **IL-1b** | -0.12 | 0.000 | -0.09 | 0.017 | 0.69 | 0.271 |
| **IL-12P70** | 2.24 | 0.011 | 1.51 | 0.053 | 0.67 | 0.122 |
| **IL-13** | 0.59 | 0.105 | 1.00 | 0.996 | 1.68 | 0.030 |
| **IL-15** | 1.41 | 0.047 | 1.10 | 0.397 | 0.78 | 0.225 |
| **IP-10** | 1.05 | 0.403 | 0.94 | 0.243 | 0.89 | 0.047 |
| **MIP-1a** | 0.40 | 0.007 | 0.45 | 0.176 | 1.11 | 0.608 |
| **MIP-1b** | 1.84 | 0.003 | 1.25 | 0.321 | 0.68 | 0.039 |
| **VEGF** | 1.31 | 0.008 | 1.26 | 0.101 | 0.96 | 0.573 |

| | | | | | | |
|---|---|---|---|---|---|---|
| **IL-1a** | 0.99 | 0.894 | 0.81 | 0.011 | 0.82 | 0.022 |
| **IL-2Ra** | 1.12 | 0.112 | 0.95 | 0.334 | 0.85 | 0.035 |
| **IL-12p40** | 0.92 | 0.119 | 0.87 | 0.043 | 0.94 | 0.283 |
| **IL-16** | 1.08 | 0.203 | 1.11 | 0.043 | 1.03 | 0.588 |
| **IL-18** | 1.87 | 0.107 | 3.23 | 0.015 | 1.72 | 0.466 |
| **MIF** | 1.08 | 0.207 | 0.93 | 0.307 | 0.86 | 0.018 |
