# Supplementary material for: Peritoneal Fluid Cytokines Reveal New Insights of Endometriosis Subphenotypes
Source: Int J Mol Sci. 2020 May 15;21(10):3515. doi: 10.3390/ijms21103515 (PMC7278942; doi:10.3390/ijms21103515)
Supplement: Supplementary file 1 [file ijms-21-03515-s001.zip › Table S1.pdf]

Table S1. List of quantified cytokines, chemokines and growth factors and their abbrevia
Detected cytokines in the peritoneal fluids are marked with ticks (✓)

No.	Name	Detected
1	EGF – Epidermal growth factor	
2	Eotaxin (CCL11) - C-C motif chemokine 11	✓
3	FGF-2 (FGF basic) - Basic fibroblast growth factor	
4	GM-CSF - Granulocyte macrophage colony-stimulating factor	✓
5	GROα (CXCL1) - Chemokine (C-X-C motif) ligand 1; Growth regulated oncogene-alpha	✓
6	HGF – Hepatocyte growth factor	✓
7	IFNγ – Interferon gamma	✓
8	IFNα – Interferon alpha	✓
9	IL-1RA – Interleukin-1 receptor alpha	✓
10	IL-1β - Interleukin-1 beta	✓
11	IL-1α - Interleukin-1 alpha	✓
12	IL-2 - Interleukin-2	
13	IL-4 - Interleukin-4	
14	IL-5 - Interleukin-5	
15	IL-6 - Interleukin-6	✓
16	IL-7 - Interleukin-7	✓
17	IL-8 (CXCL8) - Chemokine (C-X-C motif) ligand 8; Interleukin-8	✓
18	IL-9 - Interleukin-9	
19	IL-10 - Interleukin-10	✓
20	IL-12p40 - Interleukin-12 70kDa heterodimer	✓
21	IL-12p70 - Interleukin-12 40kDa monodimer	✓
22	IL-13 - Interleukin-13	✓
23	IL-15 - Interleukin-15	✓
24	IL-17A - Interleukin-17A	
25	IL-18 - Interleukin-18	✓
26	IP-10 (CXCL10) - C-X-C motif chemokine 10; Interferon gamma-induced protein 10	✓
27	LIF - Leukemia inhibitory factor	
28	MCP-1 (CCL2) - Chemokine (C-C motif) ligand 2; Monocyte chemotactic protein 1	✓
29	MIP-1α (CCL3) - Chemokine (C-C motif) ligand 3; Macrophage inflammatory protein 1-alpha	✓
30	MIP-1β (CCL4) - Chemokine (C-C motif) ligand 4; Macrophage inflammatory protein 1-beta	✓
31	βNGF - Beta nerve growth factor	✓
32	PDGF-BB - Platelet-Derived Growth Factor-BB	
33	RANTES (CCL5) - Chemokine (C-C motif) ligand 5	
34	SCF - Stem cell factor	✓

35	SDF1α (CXCL12) - C-X-C motif chemokine 12; stromal cell-derived factor α	✓
36	TNFα - Tumor necrosis factor alpha	✓
37	TNFβ (LTA) - Lymphotoxin-alpha; tumor necrosis factor-beta	✓
38	VEGF-A - Vascular endothelial growth factor A	✓
39	IL-16	✓
40	IL-3	✓
41	MCP-3	✓
42	MIF	✓
43	MIG	✓
44	SCGF-b	✓
45	TRAIL	✓
46	G-CSF	✓
47	IL-2RA	✓
48	CTACK	✓

ıtions.
