# Supplementary material for: Peritoneal Fluid Cytokines Reveal New Insights of Endometriosis Subphenotypes
Source: Int J Mol Sci. 2020 May 15;21(10):3515. doi: 10.3390/ijms21103515 (PMC7278942; doi:10.3390/ijms21103515)
Supplement: Supplementary file 1 [file ijms-21-03515-s001.zip › Table S4.pdf]

Table S4. Significantly altered cytokines in endometriosis subphenotypes by univariate statistical analysis

All Phases	OE vs EM-		PE vs EM-		DIE vs EM-	
	Fold Change	*P*-value	Fold Change	*P*-value	Fold Change	*P*-value
**IL-16**	0.79	0.102	0.65	0.024	1.04	0.816
**HGF**	0.81	0.469	0.40	0.024	0.34	0.024
**IFN-a2**	0.87	0.070	0.81	0.032	0.84	0.102
**MCP-3**	0.94	0.725	0.60	0.042	0.86	0.540
**IL-1a**	1.01	0.869	0.90	0.082	1.26	0.003
**VEGF**	1.70	0.117	1.33	0.302	2.45	0.005
**IL-12P70**	1.54	0.150	1.12	0.717	1.75	0.032

Proliferative	OE vs EM-		PE vs EM-		DIE vs EM-	
	Fold Change	*P*-value	Fold Change	*P*-value	Fold Change	*P*-value
**IL-16**	0.72	0.080	0.62	0.045	0.92	0.744
**IFN-a2**	0.83	0.051	0.77	0.026	0.84	0.230
**IL-1a**	0.93	0.297	0.94	0.441	1.43	0.009
**VEGF**	3.57	0.024	2.33	0.016	2.14	0.041
**IL-12P70**	3.54	0.003	2.38	0.004	2.68	0.001
**CTACK**	1.16	0.297	1.41	0.027	1.20	0.363
**TNF-a**	3.39	0.010	1.07	0.867	0.51	0.277
**IP-10**	1.78	0.012	1.14	0.588	1.41	0.207
**IL-15**	1.59	0.040	1.44	0.131	1.16	0.591
**IL-1ra**	1.53	0.137	0.89	0.755	0.33	0.049

Secretory	OE vs EM-		PE vs EM-		DIE vs EM-	
	Fold Change	*P*-value	Fold Change	*P*-value	Fold Change	*P*-value
**IFN-a2**	0.84	0.389	0.84	0.299	0.83	0.047
**VEGF**	0.65	0.133	0.65	0.424	2.71	0.006
**IL-12P70**	0.59	0.173	0.59	0.342	1.43	0.029
**IL-15**	0.91	0.028	0.91	0.726	1.05	0.459
**MCP-1**	0.61	0.767	0.61	0.122	1.77	0.022
